# Traditional Chinese Medicine and Western Medicine Share Similar Philosophical Approaches to Fight COVID-19

**DOI:** 10.14336/AD.2021.0512

**Published:** 2021-08-01

**Authors:** Fangfang Zhao, Zhenhong Yang, Ningqun Wang, Kunlin Jin, Yumin Luo

**Affiliations:** ^1^Institute of Cerebrovascular Disease Research and Department of Neurology, Xuanwu Hospital of Capital Medical University, Beijing 100053, China.; ^2^Department of Pharmacology and Neuroscience, University of North Texas Health Science Center, TX 76107, USA.; ^3^ Beijing Institute for Brain Disorders, Capital Medical University, Beijing 100069, China.

**Keywords:** Western medicine, Traditional Chinese medicine, COVID-19, cold-dampness, calcium, vitamin D

## Abstract

Though disciplines in the same field, modern medicine (Western medicine) and traditional medicine (Traditional Chinese medicine, TCM) have been viewed as two distinct and divergent fields of medicine and thus differ greatly in their ways of diagnosing, treating, and preventing disease. In brief, Western medicine is primarily an evidence (laboratory)-based science, whereas TCM is more of a healing art based on the theory of Yin and Yang and the five elements in the human body. Therefore, whether TCM and Western medicine could use similar philosophical approaches to treat disease remains unclear. It is well-known that vitamin D enhances immune function and reduces the spread of some viruses. Indeed, recent evidence shows that the blood calcium level is strongly associated with COVID-19 severity, and vitamin D supplementation has shown favorable effects in viral infections. According to TCM theory, the pathogenesis of COVID-19 is closely associated with cold-dampness, an etiological factor in TCM. Cold-dampness could be attenuated by sun exposure and Wenyang herbs, both of which can restore the vitamin D level in the blood in Western medicine. Therefore, TCM and Western medicine could share similar philosophical methods to fight COVID-19 and understanding their philosophical theories could achieve the maximum benefits for treatment of COVID-19 and other diseases.

Coronavirus disease 2019 (COVID-19) has been rapidly spreading worldwide for more than a year and is still not under control [1,2]. Currently, severe acute respiratory syndrome coronavirus 2 (SARS-CoV-2) vaccines may be the best way to control the COVID-19 pandemic. However, the prevention and treatment of COVID-19 remain the top priorities in the current circumstances. Undeniably, traditional medicine (Traditional Chinese medicine, TCM) has played a significant role in the treatment of COVID-19 [3]. Although TCM was recognized by the World Health Organization (WHO) for the first time in 2019 (11^th^ version of International Statistical Classification of Diseases and Related Health Problems), TCM has not been largely accepted by Western medicine (modern medicine). In fact, Western medicine and TCM have always been largely incompatible due to their distinct origins and philosophical theories. In Western medicine, the state of health clearly divides from the disease, while TCM considers health as a balanced state and disease as an unbalanced state. For example, Western medicine performs diagnosis based on laboratory tests and focuses on eliminating symptoms, while TCM diagnoses through the symptoms and appearances to seek to address the overall systemic problem. Therefore, whether TCM and Western medicine could use similar philosophical approaches to treat diseases remains vague. As each approach has values and therapeutic methods that can be explained both scientifically and philosophically, both could be mutually beneficial rather than exclusive, and we believe that TCM and Western medicine could share similar philosophical methods to fight COVID-19. Therefore, understanding TCM and Western medicine, and even a combination of TCM and Western medicine to form an integrative medicine approach may achieve the maximum benefits for the treatment of COVID-19 and other diseases.

## Treatment of COVID-19 using TCM

### TCM theory of COVID-19 pathogenesis

In TCM theory, the COVID-19 epidemic conforms to the category of “cold-dampness plague”. Among them, “warm” refers to the symptom of fever, and “epidemic” refers to the infectious and epidemic nature of the disease. Therefore, the main clinical manifestations of COVID-19 are coldness and dampness [4-7]. An analysis of 2, 831 cases of coronavirus pneumonia in COVID-19 patients found that wet symptoms accounted for over 50% of cases [8]. “Dampness poison” is a kind of pestilence, which enters the human body through the skin, nose, or mouth. Dampness poison, as the main cause, varies from person to person. It moves through the circulation, from the exterior into the interior, and gradually penetrates into the deeper parts of the body, blocking the normal physiological functions of lung and spleen. Dampness poison produces heat and a Qi deficiency adversely affects Yin. Severe cases can lead to rapid depletion of Qi and Yin, which could lead to death due to internal closure and external detachment. Therefore, dampness poison is the pathogenesis of COVID-19. Dampness poison easily encroaches on the lung, spleen, and stomach and invades the lung, causing the lung function of dispersing and descending to be abnormal and an upward inversion of the gas turbine, resulting in clinical symptoms, such as cough, chest tightness, and shortness of breath. Dampness poison trapped the spleen, resulting in yang deficiency, and Qi deficiency in the spleen and stomach could lead to clinical symptoms, including fatigue, loss of appetite, and a thick and greasy tongue coating.

### TCM treatment of COVID-19

Yang Qi is considered to be the root of life and is very important according to TCM theory. While Wenyang is the supplement to the Yang Qi of the human body by using warm methods, the cold-dampness syndrome can be treated by Wenyang to promote diuresis. Sunlight is a physical approach of Wenyang. Many decoctions of Wenyang can promote diuresis, such as Wuling powder [9]. In a study of 84 patients with COVID-19, all patients had cold or wet symptoms, and the method of Wenyang and damp-clearing is usually used in the treatment.[10]. The “Clear the lungs and relieve toxicity” formula is a prescription mainly aimed at the patients with cold-dampness, with relatively more pungent and hot drugs for Wenyang [11-15]. The principle of treatment is to strengthen the body resistance to infection.

As COVID-19 is mainly caused by cold-dampness, it is recommended to consume warm foods such as ginger, garlic, and scallions for treatment. A diet based on scallion, ginger, and jujube soup can warm the stomach and blood. Patients with weak Yang Qi can consume a Danggui ginger mutton soup. Many remedies for COVID-19 include ginger. In TCM theory, ginger enters the lung’s meridian, dispels cold and relieves the exterior, warms the lungs, stops coughing, and prevents toxic gases. Ginger has a strong inhibitor for the SARS-CoV-2 protein and the angiotensin-converting enzyme 2, the latter being a receptor used by the coronavirus to enter the body. In fact, the selectivity of ginger for the SARS-CoV-2 protein is prominent. Among the main components of ginger, 11 components could inhibit the SARS-CoV-2 [16]. Therefore, ginger can be used as a therapeutic dietary component to prevent and treat COVID-19.

External treatments include wearing sachets, moxibustion, and sun exposure. According to the cold and wet nature of the pestilence, sachets containing fragrant herbs, such as atractylodis, grass fruit, *Artemisia argyi*, Huoxiang, *Angelica dahurica*, can dispel cold and dampness and eliminate pathogens [17,18]. Moxibustion stimulates the acupoints on the body surface with the heat generated by burning moxa sticks, thereby stimulating the meridians of the body and regulating the balance of Yin and Yang to achieve a therapeutic effect. Regular moxibustion can make Yuan Qi stronger, and moxibustion can dispel cold and dampness. Therefore, moxibustion at the Shenque, Guanyuan, Zusanli, and other acupoints can disperse cold and dehumidification, and Wenyang can be used to prevent epidemic diseases. Sunlight is highly beneficial to human beings and is the basis of Wenyang treatment. In TCM theory, the back of the human body is the location of the Du Meridian, a place where Yang Qi gathers. The method of Wenyang requires sun exposure to illuminate the Du Meridian on the back, which is conceived as the legendary battery of the human body.

## Treatment of COVID-19 using Western medicine

Viral vaccines are the most effective, economical, and long-lasting methods for preventing and controlling diseases. In view of the possible common epidemic of the coronavirus and influenza virus, WHO recommends priority vaccination of the influenza vaccine, especially for health workers, the elderly, and pregnant women. However, the vaccine and the current epidemic prevention policy may be considered as complementary, as the vaccine cannot completely replace the existing prevention and control measures. Strict prevention and control remain effective measures to reduce the spread of epidemics. Therefore, it is necessary to strengthen science education and popularization of the COVID-19 vaccine. Thus far, there are no specific antiviral drugs for SARS-COV-2. Therefore, based on previous experience in the treatment of similar viruses, such as Severe Acute Respiratory Syndrome (SARS) and Middle East Respiratory Syndrome (MERS), as well as the research on SARS-COV-2 genomic information and bioinformatics, a batch of drugs for the treatment of COVID-19 were screened out from the existing antiviral drugs, including ribavirin, abidol, and α-interferon. A combination of two antiviral drugs is generally recommended, with close attention to possible side effects. With the development of genome sequencing and bioinformatics research on SARS-COV-2, it is necessary to establish a screening model for viral-infected cells to study small molecule drugs. Accumulating evidence shows that patients with COVID-19 have immune imbalances. Hence it has been suggested that the immune system of patients should be adjusted on the basis of antiviral therapy. In terms of immunotherapy, in addition to recovered plasma, patients with rapid progression and severe disease can benefit from intravenous neocoronal pneumonia human immunoglobulin.

## Exploring the link between Western medicine and TCM from the point of view of Ca^2+^

### The role of calcium in COVID-19 in Western medicine

At present, in the research on coronavirus pneumonia, hypocalcemia has been identified as a susceptible factor. Disruption of calcium homeostasis facilitates the viral control of host cells. Viral invasion of host cells can trigger a cascade of reactions, including alterations in Ca^2+^ permeability, transport, and exchange, which ultimately lead to alterations in the Ca^2+^ level, resulting in the infection, spread, and even exacerbation of the virus in humans [19]. Many cases occur in hospitals with elderly patients. The long-term lack of sun exposure can cause calcium levels to drop. Of course, hypoimmunity and various underlying diseases are also among the causes. Previous studies have shown that calcium played a central role in the transmission and replication of the SARS coronavirus, MERS coronavirus, and Ebola virus [20-22]. In a large group of SARS patients in North America, 60% had hypocalcemia on admission and 70% had hypocalcemia during hospitalization [23]. Similarly, a study on patients with an Ebola virus infection in American and European hospitals also reported a similar incidence of hypocalcemia [24].

Recently, other studies have reported a high incidence of hypocalcemia in patients with COVID-19 [25-28]. Luigi *et al*. investigated the incidence of hypocalcemia in 531 patients with COVID-19 at a single center and evaluated the possible clinical significance of their findings [29]. In univariate and multivariate analyses, hypocalcemia was identified as an independent risk factor that was highly correlated with hospitalization. In the univariate analysis, hypocalcemia was also found to be associated with mortality and the occupancy rate in intensive care units. The incidence of low calcium levels in severe or critical cases was found to be greater than that in moderate cases. Other studies found that low calcium levels were positively correlated with decreased lung function indices (such as oxygenation index and PaO_2_), decreased lymphocyte count, abnormal biochemical indices (low-density protein and C-reactive protein), and an abnormal inflammatory index [30].

Liu *et al*. investigated the incidence of hypocalcemia in patients with different severities of COVID-19 [31]. Compared with patients with moderate infection, patients with a severe COVID-19 infection were more likely to have hypocalcemia, even after adjustment for age and complications. Therefore, hypocalcemia may be an indicator of a severe stage of COVID-19. Luigi *et al*. also found that hypocalcemia was more common in men and the elderly, which may explain the fact that COVID-19 occurs more frequently in older and male patients [29]. In addition, the proportion of patients with severe hypocalcemia during hospitalization was relatively high, supporting a specific correlation with the disease. Given the high incidence of hypocalcemia in COVID-19 patients and the need for hospitalization, we suggest that Ca^2+^ levels should always be assessed during the initial hospital assessment to better identify critically ill patients. Taken together, hypocalcemia may play a role in the endocrine system and in SARS-Cov-2. Early diagnosis and treatment of hypocalcemia can reduce organ damage during the severe stage of COVID-19. Severe and critical cases of COVID-19 are characterized by a calcium homeostasis disorder. Strengthening the monitoring and timely treatment of serum Ca^2+^ levels in critically ill patients with COVID-19 is of great significance for improving prognosis. These clinical features contribute to the diagnosis and monitoring of COVID-19 patients. Since the global prevalence of COVID-19 may persist longer, it is strongly recommended that first-line physicians improve their knowledge regarding potential hypocalcemia in patients with COVID-19.

### Mechanism of hypocalcemia in COVID-19

Although no direct evidence supports that SARS-CoV-2 is related to Ca^2+^ regulation, it has been confirmed that Ca^2+^ is necessary for viral entry, viral gene replication, and viral particle maturation and release [32]. The membrane sequence of the fusion peptide of the SARS-CoV-2 glycoprotein depends on Ca^2+^, and Ca^2+^ also plays an important role in SARS-CoV-2 virus invasion [33].

Ca^2+^, as a second messenger in animals, plays an important role as an intracellular signaling ion. The disruption of intracellular Ca^2+^ balance caused by viral infection can lead to cell dysfunction and apoptosis [34]. Intracellular Ca^2+^ homeostasis damage plays an important role in SARS-CoV-2 infection and host cell replication [35]. The blocker of voltage-gated calcium channels could inhibit replication [36] or invasion of the influenza virus [37-39].

The SARS-CoV-2 gene sequence can encode transmembrane proteins with permeability to Ca^2+^, and this protein can be synthesized in large quantities in the process of infection [40,41]. Ca^2+^ flow is considered an important mechanism for regulating viral infection. In addition, the porcine delta coronavirus (PDCoV) can regulate calcium inflow, which is conducive to viral replication. The Ca^2+^ channel blocker (CCB) can significantly inhibit PDCoV infection [42].

A previous study reported that the CCB not only inhibited the replication of SARS-CoV-2 *in vitro*, but also played a role in alleviating the inflammatory response of patients [43]. There is currently a demand to develop a variety of drugs to deal with RNA viruses, such as SARS-CoV-2, because these viruses generally have high mutation rates. In summary, a calcium metabolism disorder is particularly important because it may become an independent biomarker for SARS-CoV-2 infection or other severe infections. Since levels of hypocalcemia are high in patients with COVID-19 and indicate the need for hospitalization, we recommend that Ca^2+^ levels should always be measured at the initial hospital assessment to better identify critically ill patients.

### The role of calcium in TCM

The Wenyang method in the treatment of COVID-19 acts via the regulation of Ca^2+^. Chen *et al*. found that a reinforcing kidney-activating blood prescription (warming kidney yang) regulates calcium and phosphorus metabolism, leading to increased blood calcium levels [44]. Furthermore, recent studies have been conducted to regulate calcium levels by TCM for the treatment of osteoporosis [45-47]. Therefore, calcium levels can be increased by Wenyang. One of the ways to supplement calcium is sunbathing. Westerners like to sunburn their backs, which coincides with the TCM treatment of Wenyang and sun exposure. Most TCM physicians believe that the Du Meridian is the main Yang meridian of the body, and Wenyang advocates basking with the sun on the back.

COVID-19 mortality is negatively correlated with sunlight exposure [48]. Countries near to the equator have lower mortality rates. The evidence suggests that sunlight can reduce COVID-19 mortality [49]. Vitamin D levels in the body are significantly affected by sunlight, and there is a link between vitamin D deficiency and the severity and COVID-19 mortality [50]. Vitamin D is generated when ultraviolet light from the sun irradiates the skin and transforms into active vitamin D through the liver and kidney, which in turn promote the absorption of calcium in the human intestine. An imbalance in Ca^2+^ homeostasis can lead to the invasion of SARS-CoV-2. The absorption of Ca^2+^ cannot be separated from that of vitamin D. The activity of vitamin D is stimulated by ultraviolet light in the sun; therefore, calcium absorption in humans requires sun exposure. Advanced age is a risk factor for vitamin D deficiency. With increasing age, the absorption and synthesis of vitamin D are decreased. It also explained COVID-19 in the elderly patients with high incidence and severity of the reasons from this aspect. Previous studies have shown that vitamin D supplementation reduces the risk of COVID-19 infection and death [51], which has potential significance for the prevention of COVID-19 [52].

## Summary

Based on TCM theory, cold-dampness is the pathogenesis of COVID-19 [53]. Cold-dampness attacks the surface of the lung and closes it, trapping the spleen, and injuring Yang. Therefore, pungent warming treatments to dispel coldness, clear the lung, and remove pathogens is recommended. At the same time, attention should be paid to invigorate the spleen, remove dampness, and resolve phlegm to promote recovery of the spleen and hinder the replication of pathogens cannot. Second, cold-dampness can easily hurt Yang. Hence, the treatment should consider the body’s Yang and include the cautious application of bitter cold medicine. Importantly, lingering diseases and changing syndromes may present additional complications. Therefore, physicians need to adjust to the actual situation, identify the main contraindications, provide prescriptions considering the time, place, and person, and use different methods at different stages of disease. Pharmacological analysis has shown that these drugs have strong antiviral and antibacterial properties and are effective in strengthening the immune system.

In summary, COVID-19 is mainly caused by cold-dampness, which causes cold pathogens to attack yang and heat pathogens to attack yin, a process that severely weakens the patient’s system after a long course of the disease. Therefore, attention should be paid to diet and lifestyle after the disease, and Wenyang should nourish yin as appropriate to prevent the entry of pathogenic gases. Acupuncture, sunbathing, and other comprehensive therapies can significantly improve the symptoms. In addition, the advantage of TCM intervention is that it allows the immune function of the human body to adjust, stimulates the body’s own ability to resist disease, eliminates pathogenic factors, and strengthens the vital energy. Under this type of regimen, patients with mild infection tend to recover and patients with moderate infection can avoid further complications and stop the progression of the disease. Nevertheless, the global epidemic of COVID-19 is still severe. Considering the efficiency of TCM on pneumonia and other complications, it is expected to play a broader and more in-depth role in the prevention and treatment of COVID-19 in the future.

Bruce Elwald, a senior adviser to the Director- General of the WHO, stated that China's approach is the proven effective method [54]. At present, more than 200 countries worldwide have experienced COVID-19 outbreaks. In the absence of specific drugs, the experience of China and the application of TCM drugs can be used as valuable references. In TCM theory, the main strategy consists of enhancing health while removing toxins and treating the cold syndrome with Wenyang. In terms of prevention, sunbathing and diet therapy play an important role in increasing the levels of calcium ions. TCM also has great potential to treat COVID-19 by improving calcium levels through the Wenyang method. Furthermore, sun exposure and use of ginger are beneficial approaches for treating cold symptoms for people with a cold constitution. TCM is a theoretical system developed through thousands of years of practice in the form of the black-box theory, and new knowledge is constantly introduced and revised in practice. It is a natural medical system that is related to human health and disease. Although there are many factors that need to be interpreted and understood in light of the scientific methods, TCM treatments have been proven effective for several conditions. However, further evidence is needed to establish the usefulness of TCM treatments for the management of COVID-19.
